# A *d* factor? Understanding trait distractibility and its relationships with ADHD symptomatology and hyperfocus

**DOI:** 10.1371/journal.pone.0292215

**Published:** 2023-10-25

**Authors:** Han Zhang, Akira Miyake, Jahla Osborne, Priti Shah, John Jonides

**Affiliations:** 1 Department of Psychology, University of Michigan, Ann Arbor, Michigan, United States of America; 2 Department of Psychology and Neuroscience, University of Colorado Boulder, Boulder, CO, United States of America; University of Hertfordshire, UNITED KINGDOM

## Abstract

People differ substantially in their vulnerability to distraction. Yet, many types of distractions exist, from external stimulation to internal thoughts. How should we characterize individual differences in their distractibility? Two samples of adult participants (total *N* = 1220) completed a large battery of questionnaires assessing different facets of real-world distractibility. Latent modeling revealed that these measures could be explained by three correlated-yet-distinct factors: external distraction, unwanted intrusive thoughts, and mind-wandering. Importantly, about 80% of the total variance in these three factors could be explained by a single higher-order factor (*d*) that could be construed in terms of a person’s general distractibility, and this general distractibility model was replicated across the two samples. We then applied the general distractibility model to understand the nature of ADHD symptomatology and hyperfocus (an intense state of long-lasting and highly focused attention). *d* was substantially associated with self-reported ADHD symptoms. Interestingly, *d* was also positively associated with hyperfocus, suggesting that hyperfocus may, to some degree, reflect attention problems. These results also show marked consistencies across the two samples. Overall, the study provides an important step toward a comprehensive understanding of individual differences in distractibility and related constructs.

## Introduction

Distraction is prevalent in our daily lives. As you read this article, you might be distracted by the voices of people around you, by worries about an impending decision on a grant application, or by fantasies about your next holiday travel plan.

Distraction has been a central topic in the study of cognition for well over 100 years [1]. In this literature, one central theme has been that people differ substantially in how easily they get distracted. Numerous studies have shown that people who report being distracted more easily are at a higher risk of poor performance in school and work settings [2, 3] and even sometimes prone to serious accidents [4]. The issue of interindividual variability also cuts across multiple forms of psychopathology, most notably as a symptom of attention-deficit/hyperactivity disorder (ADHD) but also featured in other psychiatric disorders including anxiety, depression, and schizophrenia [5–9]. Thus, understanding individual differences in distractibility is important not only for predicting for whom attention is likely to fail but also for understanding the cognitive underpinnings of psychopathology.

How can we best characterize individual differences in distractibility? Surprisingly, there is no clear, comprehensive answer to this question yet, because there are multiple different facets to distractibility. Distractibility can be conceptualized to mean susceptibility to irrelevant stimulation [5], the tendency to have thought intrusions [10], the tendency to experience repetitive negative thinking [11], or the tendency to engage in mind-wandering [12]. Oftentimes, however, these different conceptualizations of distractibility have been studied in isolation; in fact, most existing studies on this topic consider only a single aspect of distractibility (e.g., external distraction) and often assess it with a single measure. Moreover, studies on distractibility are often conducted with underpowered samples (most often with just convenience samples of college students) and without replication of the results. For these reasons, it is yet unclear whether and to what extent these constructs reflect different facets of distraction.

The current study used an individual-differences approach to establish a structure of distractibility. We assessed different facets of distractibility in two large samples (one drawn from college students and the other drawn from the broader community) by administering a large battery of self-report measures, each facet represented by multiple questionnaires. Using confirmatory factor analyses, we specified how these constructs are empirically related to one another at the latent level. Then, we applied our resulting model to understand individual differences in ADHD symptomatology as well as hyperfocus, a prolonged state of concentration often observed among individuals with high levels of ADHD symptomatology [13]. As such, the current study sheds new light not only on the factor structure of individual differences in different types of distractibility but also on the nature of distractibility associated with ADHD.

### Different forms of distraction

The term “distraction” is commonly used to refer to irrelevant percepts in the external environment, such as irrelevant visual stimuli (e.g., a visually salient object unrelated to a current visual search) and irrelevant auditory stimuli (e.g., irrelevant speech). The study of external distraction has a long and distinguished history in the field of psychology featuring experimental manipulations of external stimulation [14–16]. Furthermore, the notion of distractibility as an important individual-differences factor has long been proposed, but “distractibility” in these early studies almost exclusively refers to distraction by external stimulation [5, 17–20]. However, external stimulation is likely not the only source of distraction; there is increasing evidence showing that performance can be hampered by information that is hypothesized to have an internal origin [21–23]. Three notable constructs that are conceptually distinguishable from external distraction are (*a*) thought intrusions, (*b*) repetitive negative thinking, and (*c*) mind-wandering.

*Thought intrusion* is a common experience. Studies have found that over 80% of healthy individuals experience some form of intrusive thoughts [24]. The contents of these thoughts are often negative (e.g., an unpleasant memory) and sometimes even abhorrent and disgusting (e.g., violence against someone). Though typically studied from a psychopathology perspective, thought intrusions constitute a form of cognitive distraction because they interrupt the flow of thought and capture mental capacity. As a result, one of the hallmarks of thought intrusions is that they interfere with ongoing task performance [25].

*Repetitive negative thinking* is defined as “repetitive thinking about one or more negative topics that is experienced as difficult to control” [26, p.193]. Two common forms of repetitive negative thinking are rumination, defined as past-oriented persistent dwelling on causes and consequences of one’s distress [27], and worry, defined as future-oriented repetitive thinking about potential threats, uncertainties, and risks [28, 29]. Both rumination and worry are associated with difficulty concentrating and low task performance [21, 23, 30]. Although repetitive negative thinking and thought intrusions share many similarities (e.g., unpleasant, distracting, and uncontrollable), there are some fine-grained differences between them. Clark and Rhyno [24] suggest that repetitive negative thinking may represent a more persistent form of cognitive interference, whereas thought intrusions may be more sudden, undirected, and somewhat unexpected (akin to a “mental flash”).

The term *mind-wandering* was used by Smallwood and Schooler [12] to describe a collection of terms that all entail a spontaneous shift of attention away from a task toward “unrelated inner thoughts, fantasies, feelings, and other musings” (p. 946). The term has since been employed to characterize a diverse array of mental phenomena, including intentional or deliberate forms of mind-wandering (as opposed to unintentional or spontaneous), and unguided thoughts that may arise even in the absence of any task [31]. Considering the expansive usage of the term, not all mental phenomena grouped under it can be deemed as distractions. In the study we report here, we narrows its focus on the facet of mind-wandering that is spontaneous and unrelated to the task at hand. Under this definition, mind-wandering constitutes a form of distraction in many cognitive tasks [32].

In conception, for several reasons, mind-wandering is not considered as simply repetitive negative thinking or thought intrusion. First, mind-wandering is not necessarily negative or unpleasant. For example, during a lecture, students may find themselves thinking about unrelated things such as their plans for the weekend. While these thoughts disrupt attention to the lecture, they may be quite pleasant, interesting, and constructive for other purposes [33]. Second, mind-wandering does not necessarily involve recurrent, repetitive, or cyclical mental content [34]. A person may meander from topic to topic during an episode of mind-wandering; different mind-wandering episodes may also involve wholly different topics. Third, individuals are often unaware of their mind-wandering state [35] whereas repetitive negative thinking and thought intrusion are often associated with conscious appraisals and attempts to resist [36].

### The relationships among facets of distractibility

We have reviewed several constructs that capture different aspects of how easily a person can be distracted, but how exactly are they related to each other? That is, if a person is prone to one type of distraction, are they also prone to other types of distractions? To our knowledge, no prior work has examined the relationships among all of these constructs simultaneously within a single study. Without an empirical assessment of their relationships, we run the risk of *jingle-jangle* fallacies [37, 38]. In the jingle fallacy, the same term is used to refer to distinct constructs, whereas in the jangle fallacy, different terms are used to refer to the same construct.

At one extreme, for example, external distraction, thought intrusions, repetitive negative thinking, and mind-wandering may be completely separable and not share anything in common, thus making the practice of calling them all instances of distraction highly misleading (representing a case of the jingle fallacy). At the other extreme, they may be completely redundant constructs (thus, representing a case of the jangle fallacy) so there is no need to differentiate among them.

Both of these accounts are unlikely, however, in light of previous studies examining the relationship between external distraction and mind-wandering, which have shown that they are correlated yet distinct constructs [39, 40]. For example, Unsworth and McMillan [40] obtained self-reports of external distraction and mind-wandering by presenting thought probes as participants completed laboratory tasks. Using confirmatory factor analysis, they found that a two-factor model consisting of an external distraction factor and a mind-wandering factor fit statistically better than combining them into one factor (i.e., constraining their correlation to 1), although there was a sizable correlation between the two factors (*r* = .44). While these studies show that external distraction and mind-wandering demonstrate some overlap, they did not distinguish mind-wandering from similar constructs like thought intrusions and repetitive negative thinking. Instead, “mind-wandering” was used generally to represent distraction by internal stimuli [40].

Indeed, the practice of partitioning distraction into an external-internal dichotomy is prevalent in the literature [41–44]. By this account, thought intrusions, repetitive negative thinking, and mind-wandering should be highly correlated (if not redundant) and load onto the same factor because, in conception, they all reflect distraction by internal thoughts. However, studies have shown that mind-wandering can be experienced as quite pleasant even though it may reduce task performance [33]. Furthermore, several studies have shown that self-reported mind-wandering tendency is only moderately correlated with self-reported thought intrusions (*r*s = .24 to.36 [45, 46]). These results suggest that what is perceived as mind-wandering might not be perceived as repetitive negative thinking (or thought intrusion). If so, then a simple external-internal dichotomy may not adequately capture the relationships among these constructs.

### A general distractibility factor?

The studies reviewed above suggest that external distraction, thought intrusions, repetitive negative thinking, and mind-wandering may have some underlying commonality but also demonstrate some separability, a pattern similar to that observed in individual differences studies of executive functions [47, 48]. This pattern of “unity and diversity” provides a useful way to think about the factor structure of distractibility. It may be plausible to assume that the correlations among various forms of distraction reflect an overarching trait that governs a person’s general tendency to be distracted by irrelevant information. Additionally, there might be additional factors that determine a person’s vulnerability to a specific form of distraction. This unity/diversity specification of distractibility seems to map well onto existing theories [9, 49–53]. For example, it has been argued that a person’s domain-general working memory capacity plays an overarching role in maintaining the task goal and avoiding distractions [51]. Other specific factors, such as current life concerns, play an additional role in determining the occurrence of mind-wandering.

Several recent studies have attempted to establish a general trait of distractibility, most notably those conducted by Forster and Lavie [54, 55]. These authors created a laboratory task that was designed to parallel external distractions in daily life. In this task, external distraction was introduced by the presence of irrelevant cartoon figures in a visual search task. The dependent measure was a distractor-interference score based on the RT difference between distractor-absent and distractor-present conditions. Forster and Lavie [54, 55] found that the distractor interference score was correlated with a self-reported measure of mind-wandering (*r* = .26 (Exp. 1) and *r* = .38 (Exp. 3)) and with a self-reported measure of childhood ADHD symptoms (*r* = .32 (Exp. 1) and *r* = .32 (Exp. 2)). Based on these results, Forster and Lavie [55] suggested that a general factor exists that “confers vulnerability to irrelevant distraction across the general population” (p. 209). However, a subsequent study failed to replicate those findings [56]. Of particular concern is the low internal consistency of the cartoon-distraction task (.08—.26 as reported in [56]), which suggests that the task might not reliably capture individual differences in external distractibility.

In another study, Hobbiss et al. [57] assessed the degree to which people experience external distractions and mind-wandering while engaging in a wide range of daily life activities. Hobbiss et al. [57] found that self-reports of external distraction were correlated with self-reports of mind-wandering and that these measures loaded on a single factor. However, because there were seven external distraction items and a single mind-wandering item, the resulting single factor still largely captured external distraction rather than what is common to external distraction and mind-wandering.

Overall, although these recent studies provided some initial evidence for a potential higher-order distractibility factor, the evidence is still limited. Moreover, in any of the studies just reviewed here, two other forms of distraction—thought intrusions and repetitive negative thinking—were not considered. Thus, it is necessary to further investigate the notion of a general distractibility factor in a more comprehensive manner.

### Linking distractibility to ADHD symptoms and hyperfocus

By extracting a general distractibility factor, one can directly examine how the unity and diversity components of distractibility are related to other individual-differences variables. This is important because, if a variable of interest is related to multiple correlated factors, this relationship could be driven by just the general component or both the general and the specific components. We will take advantage of this feature to understand individual differences in behavioral symptoms and functional impairments associated with ADHD as well as hyperfocus.

#### ADHD symptoms and associated functional impairments

Heightened distractibility has often been associated with ADHD, defined as an ongoing pattern of inattention and/or hyperactivity-impulsivity that interferes with functioning or development [58]. Though only about 4% of adults have a formal diagnosis of ADHD, ADHD symptoms are prevalent in the general population, and a formal diagnosis represents the extreme end of a continuous distribution [59, 60]. ADHD symptoms are associated with functional impairments in various life domains, including school, work, and interpersonal relationships [61–63]. One of the diagnostic criteria of ADHD is “being often easily distracted by extraneous stimuli (for older adolescents and adults, may include unrelated thoughts)” [58].

What is the nature of the relationship between distractibility and ADHD? The separate associations between ADHD symptomatology and different forms of distractions may, in fact, be driven by a single factor that represents an individual’s overall distractibility level. However, it has been difficult to assess the role of a general factor in explaining ADHD symptoms because most studies have examined a single form of distraction in relation to ADHD.

Much of the research on distractibility and ADHD has focused on external distraction [15, 64–66], most likely because “distraction” has traditionally been studied in terms of distraction by extraneous stimuli. However, separate studies have reported that ADHD symptomatology is also associated with vulnerability to unwanted intrusive thoughts [67] and spontaneous mind-wandering [41, 43, 68]. Because different forms of distraction were not considered simultaneously in these studies, it remains difficult to specify the nature of these observed correlations.

To what extent are these correlations driven by a common factor that is elevated among people who have high levels of ADHD symptomatology? Is there anything special between ADHD and certain forms of distraction beyond general distractibility? One way to address these questions is to assess whether the relationship between a particular form of distraction and ADHD still holds after partialing out a possible general factor. If there is a special relationship between a particular form of distraction and ADHD symptoms, one should expect a meaningful relationship even after controlling for the general factor.

#### Hyperfocus

The term *hyperfocus* is used to describe episodes of long-lasting and highly focused attention that people with high levels of ADHD symptomatology often report [69, 70]. For example, although these individuals often have difficulties paying attention in lectures or getting things in order, they may find themselves spending hours writing computer programs, watching TV, and getting engrossed in creative thoughts, to the extent that they completely lose track of time or forget their personal needs. The term has also attracted huge interest in the general public with frequent discussions in the media [71–73]. Despite this interest, the nature of hyperfocus remains poorly understood.

At face value, distractibility and hyperfocus seem to be opposite constructs. The former entails a state of distracted attention, whereas the latter entails a state of deeply focused attention. Thus, it seems plausible that distractibility and hyperfocus are not correlated at all or even negatively correlated. However, there are reasons to believe that distractibility is positively correlated with hyperfocus. For example, people might be distracted by the same sorts of subjects on which they might tend to hyperfocus. Indeed, hyperfocus is sometimes (but not always) experienced as negative, such as wasting time on unimportant activities, having difficulty switching to more urgent tasks, and getting “stuck” on small details [13]. If so, then those who have a higher tendency to engage in hyperfocus might be more easily getting stuck on subjects that distract them.

An even more intriguing possibility is that hyperfocus is differentially associated with different facets of distractibility. Many instances of hyperfocus experiences include common qualities such as distorted time perception, failure to attend to the world, ignoring personal needs, and feelings of total engrossment [13]. These descriptions seem to be antagonistic to external distraction, which presumably involves engaging in some sensory processing. Similarly, it is plausible to think that one is less capable of remaining in a hyperfocus state if unwanted thoughts frequently intrude into their current train of thought. In contrast, various cases of hyperfocus seem to share certain similarities with those of mind-wandering. Mind-wandering has been viewed as a state of “decoupled attention”, which entails an inward shift of attention to one’s thoughts and feelings and, as a result, an attenuation of sensory processing [12, 35].

As such, hyperfocus seems to share some similarities with mind-wandering that are not shared with other forms of distraction. Then, general distractibility alone may not sufficiently explain the nature of hyperfocus; it may have more nuanced relationships with specific components of distractibility beyond what could be accounted for by a possible general factor.

### The current study

There were two primary goals for the current study. The first goal was to understand the relationships among several popular constructs that tap into distractibility. These constructs included external distraction, thought intrusions, repetitive negative thinking, and mind-wandering. To our knowledge, no prior work has examined the relationships among all of these constructs simultaneously within a single study. Using latent variable analyses, we assessed the extent to which these constructs, each measured by multiple instruments, are redundant or separable at the latent level. We also examined the plausibility of extracting a general factor to capture the commonality underlying these constructs.

The second goal of the study was to apply the model of distractibility derived from the study to understand the nature of ADHD symptomatology and hyperfocus. We hypothesized that the putative general factor should account for substantial variance in self-reported symptoms of ADHD and associated functional impairments. But it remains to be seen whether it has any meaningful relationship with hyperfocus. In addition to examining the role of the general factor, we were also interested in assessing the role of construct-specific variances in explaining ADHD symptoms, functional impairments, and hyperfocus. To do so, we examined how much additional variance the specific distractibility factors could explain over and above the general factor.

We addressed these two goals from a trait perspective by using questionnaires that measure individuals’ tendencies to experience various forms of distraction in daily life. Recent studies suggest that, compared to task-based (behavioral) measures, self-report questionnaire-based measures tend to converge better and be more associated with real-life behaviors [74–76]. This does not necessarily indicate that questionnaires are superior to tasks in terms of measuring distractibility, but establishing the relationships among a range of self-reported real-world distractions appears to be a reasonable starting point toward a more comprehensive understanding of distractibility. Although specifying the extent of convergence between task-derived and questionnaire-derived factors of distractibility is an important question, it is beyond the scope of the present study.

Given the exploratory nature of our research goals, we conducted an internal replication. Specifically, we collected data from two large samples (*N*s = 651 and 569) and used the first sample for exploratory analyses and the second sample for replication. The two samples differed in several dimensions, including age, gender distribution, racial composition, and occupation. As such, the results of the second sample help evaluate the replicability of our findings.

## Methods

Below we report how we determined our sample size and, all data exclusions and measures in the study [77]. All data, code, and study materials are available at https://osf.io/8j6p4/.

The procedure was approved by University of Michigan Health Sciences and Behavioral Sciences Institutional Review Board (IRB-HSBS), study ID: HUM00066883. Because both samples were collected online, written consent could not be obtained from the participants. Instead, we presented a digital version of the consent form at the beginning of the study and offered participants to select whether they agree to participate or not. Only those who provided their agreement were then given access to the full study.

### Participants

For both samples, we recruited as many participants as possible given the resources available. It is worth noting that the study was conducted from October 2020 to February 2021, a period during which COVID-19 cases were rapidly increasing in the US and in-person testing was not feasible. Therefore, both samples were recruited using online platforms.

#### Sample 1

We recruited participants from Prolifc.co that met the following criteria: (*a*) between 18 and 35 years of age; (*b*) residing in the U.S. or Canada; (*c*) native speakers of English; (*d*) with 50–3000 previous submissions and 95%-100% approval rate on the Prolific.co platform. Participants were compensated with $3.67 for participation. Data were discarded if participants failed 2 or more out of the 4 attention-check questions interspersed throughout the questionnaires (*n* = 5). We further removed data from 1 participant who had a duplicate IP address. The final sample size was 651, with a mean age of 26.9 (SD = 5.0), 45.3% female, 67.7% white, and 33.9% current students.

#### Sample 2

We recruited 615 students from the introductory psychology subject pool at the University of Michigan. Participants completed the study remotely and received partial course credit for participation. Data were discarded if participants failed 2 or more out of the 4 attention check questions (*n* = 46). Thus, the final sample size was 569, with a mean age of 18.8 (SD = 1.2), 66.3% female, and 56.8% white.

### Measures

A battery of commonly used self-report instruments was administered to assess a person’s vulnerability to external distraction, thought intrusions, repetitive negative thinking, and mind-wandering. Example items of each measure can be found in Table 1.

**Table 1:** Scale names and example items.

Scale	Example Item
*External Distraction*	
Imaginal Processes Inventory—Distractibility	I find it difficult to concentrate when the TV or radio is on.
Attentional Style Questionnaire—External	I have trouble concentrating when there is movement in the room I am in.
Attentional Control—Distraction	I have difficulty concentrating when there is music in the room around me.
*Thought Intrusions*	
White Bear Suppression Inventory	There are thoughts that keep jumping into my head.
Thought Control Ability Questionnaire	It is very easy for me to stop having certain thoughts.
Thought Suppression Inventory	I have thoughts which I would rather not have.
*Repetitive Negative Thinking*	
Perseverative Thinking Questionnaire	The same thoughts keep going through my mind again and again.
Penn State Worry Questionnaire	I am always worrying about something.
Ruminative Response Scale—Brooding	[How often do you] think “What am I doing to deserve this?”
*Mind*-*Wandering*	
Imaginal Processes Inventory—Mind-wandering	During a lecture or speech, my mind often wanders.
Mind-wandering—Spontaneous	I find my thoughts wandering spontaneously.
Daydreaming Frequency Scale	I lose myself in active daydreaming [frequency option].
*ADHD*-*related Scales*	
Adult ADHD Self-Report Scale	How often do you have problems remembering appointments or obligations?
Functional Impairments	[ADHD symptoms affect my ability to function] In my work or occupation.
Adult Dispositional Hyperfocus Questionnaire	Generally, when I am busy doing something I enjoy or something that I am very focused on, I tend to completely lose track of time.

#### Imaginal Processes Inventory—Distractibility (IPI-D)

The Imaginal Processes Inventory—Distractibility subscale is a 5-item scale originally designed by Singer and Antrobus [78] and later refined by Giambra [79] to measure task distractibility with competing stimulation. Items were rated on a 0 (definitely not true for me) to 4 (very true for me) scale. The final score was the average score of the 5 items, with a higher score indicating greater vulnerability to external distraction.

#### Attentional Style Questionnaire—External (ASQ-E)

The Attentional Style Questionnaire measures the ability to maintain attention on task-related stimuli and not to be distracted by interfering stimuli [80]. The external distraction subscale has 5 items measuring susceptibility to external interfering stimuli. Items were rated on a 1 (in total disagreement) to 6 (in total agreement) scale. We administered the full scale but used only the external subscale in data analysis because the Internal subscale was not designed to measure internal distractions specifically (e.g., “It is hard for me to stay on one activity for a whole hour.”). The final score was the average score of the 5 items in the external subscale. A higher score indicated greater vulnerability to external distraction.

#### Attentional Control—Distraction (AC-D)

The Attentional Control—Distraction scale is a 4-item scale measuring susceptibility to external distraction [81]. Items were rated on a 1 (almost never) to 5 (always) scale. The final score was the average of the 4 items, with a higher score indicating greater vulnerability to external distraction.

#### White Bear Suppression Inventory (WBSI)

The White Bear Suppression Inventory is a 15-item scale measuring the control of thoughts [82]. Items were rated on a 1 (strongly disagree) to 5 (strongly agree) scale. The final score was the average score of all items, with a higher score indicating greater vulnerability to thought intrusions.

#### Thought Control Ability Questionnaire (TCAQ)

The Thought Control Ability Questionnaire is a 20-item scale measuring one’s ability to control unwanted thoughts [83]. Items were rated on a 1 (strongly disagree) to 5 (strongly agree) scale. To make the directionality of this scale consistent with that of the other scales, we reversed the original scoring scheme so that a higher score indicated worse thought control ability. The final score was the average score of all items.

#### Thought Suppression Inventory (TSI)

The Thought Suppression Inventory is an 18-item scale measuring thought suppression [84]. Items were rated on a 1 (strongly disagree) to 5 (strongly agree) scale. Previous research has identified three subscales: intrusion, suppression attempt, and effective suppression [85]. We administered the full scale but excluded items in the suppression attempt subscale from data analysis because a suppression attempt can be either successful or unsuccessful, making it an ambiguous measure of thought suppression. The final score was the average score of all the intrusion items and the reverse-coded effective suppression items. A higher score indicated greater thought intrusion.

#### Perseverative Thinking Questionnaire (PTQ)

The Perseverative Thinking Questionnaire is a 15-item clinical measure of the extent to which individuals experience repetitive, uncontrollable, and attention-consuming thoughts [86]. Items were rated on a 0 (never) to 4 (almost always) scale. The final score was the average score of the 15 items. A higher score indicated a greater tendency to engage in repetitive negative thinking.

#### Penn State Worry Questionnaire (PSWQ)

The Penn State Worry Questionnaire is a 16-item clinical measure of excessive and uncontrollable worry [87]. Items were rated on a 1 (not at all typical of me) to 5 (very typical of me) scale. The final score was the average of the 16 items, with a higher score indicating a greater tendency to worry.

#### Ruminative Response Scale—Brooding (RRS-B)

The Ruminative Response Scale (short form) is a 10-item clinical measure of the reflection and brooding aspects of rumination [88]. The reflection subscale and brooding subscales each have 5 items. Participants were asked to read each item and indicate the extent to which they think or do each one when they feel down, sad, or depressed. Items were rated on a 1 (almost never) to 4 (almost always) scale. Previous research has shown that the brooding subscale is more representative of maladaptive thinking whereas the reflection subscale captures a less maladaptive form of rumination [88]. Thus, the brooding subscale was used as the manifest variable of repetitive negative thinking, rather than the full scale. The final score was the average of the 5 items in the brooding subscale, with a higher score indicating a greater tendency to engage in brooding.

#### Imaginal Processes Inventory—Mind-wandering (IPI-MW)

The Imaginal Processes Inventory—Mind-wandering subscale is a 6-item scale proposed by [79] using items originally from the Imaginal Processes Inventory [78]. The IPI-MW measures task-unrelated thoughts during tasks. Items were rated from 0 (definitely not true for me) to 4 (very true for me). The final score was the average of the 6 items, with a higher score indicating a greater tendency to engage in mind-wandering.

#### Mind-wandering—Spontaneous (MW-S)

The Mind-wandering—Spontaneous scale is a 4-item scale measuring spontaneous mind-wandering [81]. Items were rated on a 1 (rarely) to 7 (a lot) scale except for the 3rd item (“It feels like I don’t have control over when my mind wanders.”), which was rated on a 1 (almost never) to 7 (almost always) scale. The final score was the average of the 4 items, with a higher score indicating a greater tendency to engage in spontaneous mind-wandering.

#### Daydreaming Frequency Scale (DDFS)

The Daydreaming Frequency Scale is a 13-item scale originally from the Imaginal Processes Inventory [78]. The original scale has 12 items, but according to the factor analysis by [79], an additional item (“*I tend to get pretty wrapped up in my daydreaming*.”) should belong to this scale. Items were rated on a 1-to-5 scale. The final score was the average of all items, with a higher score indicating a greater tendency to engage in daydreaming.

#### Adult ADHD Self-report Scale

The Adult ADHD Self-report Scale is a popular 18-item clinical measure for screening adults with ADHD [89]. The 18 items correspond to the eighteen criteria used to diagnose ADHD. Each question was rated on a 5-point scale from 0 (Never) to 4 (Very often). The scale uses a dichotomous-scoring system such that responses to each question are dichotomized into whether a particular symptom is present or not. For example, for the question “How often do you have problems remembering appointments or obligations?”, a response of “sometimes”, “often”, or “very often” indicates the presence of the symptom, whereas a response of “never” and “rarely” indicates the absence of the symptom. The total score was calculated by counting all positive symptoms, yielding a theoretical range of 0–18.

#### ADHD functional impairment questionnaire

The functional impairment questions were selected from the Current Symptoms Scale developed by [90]. The questionnaire includes 10 questions regarding functional impairment in ADHD. The purpose of this scale is to understand how ADHD symptoms potentially impact different areas of an individual’s life (i.e., school, work, relationships). These questions were presented immediately following the Adult ADHD Self-report Scale. Participants were asked to indicate “To what extent do the problems you may have selected on the previous questionnaire interfere with your ability to function in each of these areas of life activities?” Each item has the following response options: “never/rarely”, “sometimes”, “often”, and “very often”. These items were rated on a 0-to-3 scale. The final score was the average score of the 10 items, with a higher score indicating greater functional impairment.

#### Adult Dispositional Hyperfocus Questionnaire

The adult dispositional hyperfocus questionnaire is a 12-item scale measuring one’s tendency to experience hyperfocus [13], a state of heightened, focused attention that individuals with ADHD frequently report. Each item has the following response options: “never”, “1–2 times every 6 months”, “1–2 times per month”, “once a week”, “2–3 times a week”, and “daily”. These items were rated on a 1-to-6 scale (1 corresponding to “never” and 6 corresponding to “daily”). The final score was the average rating of all items, with a higher score indicating a greater tendency to engage in hyperfocus.

### Procedure

Questionnaires were presented to the participants in a pseudo-random order such that those of the same category were always presented together. The presentation order within each category was randomized for each participant, except that the Functional Impairment Questionnaire was always presented immediately after the Adult ADHD Self-report Scale.

### Data analysis

Our data analytic plan consisted of two main steps. First, we conducted a series of confirmatory factor analyses to explore the factor structure of distractibility. Second, in structural equation modeling, we related our models of distractibility to individual differences in ADHD symptoms, functional impairments, and hyperfocus. Throughout the analyses, we used Sample 1 for exploratory purposes and Sample 2 for replication.

Data analysis was conducted in the *R* environment. Latent factor modeling was conducted using the *lavaan* package [91] with robust maximum likelihood, which produces the Satorra-Bentler *χ*^2^ statistic (S-B *χ*^2^) for the overall model fit. A non-significant *χ*^2^ value is often desirable, but with a large sample, even trivial mis-specifications can produce a significant result. For this reason, we supplemented the *χ*^2^ statistic with additional fit indices, such as the comparative fit index (CFI), the robust root mean square error of approximation (RMSEA), and the standardized root mean square residual (SRMR). By convention, the fit of a model is deemed acceptable when CFI > .90, RMSEA < .08, and SRMR < .08. For RMSEA, we also report the 90% confidence interval (90% CI) to assess how close it is to the boundary of misfit.

For model comparison, we report the S-B *χ*^2^ difference test and the Bayes Factors (*BF*s) derived from the BIC approximation [92]. Following recommendations by Raftery [93], we consider 1 < *BF* <= 3 to be weak supporting evidence, 3 < *BF* <= 20 to be positive supporting evidence, 20 < *BF* <= 150 to be strong supporting evidence, and *BF* > 150 to be very strong supporting evidence. Conversely, we consider 1/3 <= *BF* < 1 to be weak evidence against, 1/20 <= *BF* < 1/3 to be positive evidence against, 1/150 <= *BF* < 1/20 to be strong evidence against, and *BF* < 1/150 to be very strong evidence against.

## Results

Table 2 presents the descriptive statistics for all measures for both Samples 1 and 2. Overall, all measures had satisfactory reliability values and normal levels of skewness and kurtosis in both samples. Compared to Sample 1, the standard deviations associated with Sample 2 were consistently lower, suggesting that Sample 2 was more homogeneous, which is consistent with the fact that they were college students enrolled in an introductory psychology course.

**Table 2:** Descriptive statistics for all measures analyzed in the current study.

Scale Name	*Sample* 1					*Sample* 2				
	mean	*SD*	skew	kurtosis	alpha	mean	*SD*	skew	kurtosis	alpha
*External Distraction*										
Imaginal Processes Inventory—Distractibility	2.30	0.92	-0.29	-0.35	0.85	2.45	0.79	-0.09	-0.26	0.78
Attentional Style Questionnaire—External	3.43	1.06	0.00	-0.10	0.81	3.58	0.99	0.03	-0.40	0.78
Attentional Control—Distraction	2.76	1.04	0.19	-0.84	0.88	3.04	0.97	0.03	-0.83	0.84
*Thought Intrusions*										
White Bear Suppression Inventory	3.56	0.77	-0.57	0.29	0.92	3.54	0.69	-0.26	-0.15	0.90
Thought Control Ability Questionnaire	3.22	0.83	-0.15	-0.48	0.95	3.19	0.71	-0.02	-0.51	0.93
Thought Suppression Inventory	3.20	0.80	-0.05	-0.25	0.91	3.16	0.69	0.16	-0.41	0.89
*Repetitive Negative Thinking*										
Perseverative Thinking Questionnaire	2.11	0.96	-0.22	-0.46	0.97	2.16	0.80	0.04	-0.36	0.95
Penn State Worry Questionnaire	3.37	0.98	-0.27	-0.79	0.96	3.56	0.86	-0.37	-0.55	0.95
Ruminative Response Scale—Brooding	2.39	0.78	0.19	-0.80	0.85	2.41	0.72	0.28	-0.76	0.80
*Mind*-*Wandering*										
Imaginal Processes Inventory—Mind-wandering	2.15	0.95	0.03	-0.67	0.89	2.17	0.87	0.17	-0.71	0.86
Mind-wandering—Spontaneous	4.29	1.50	-0.29	-0.56	0.91	4.21	1.35	-0.03	-0.41	0.88
Daydreaming Frequency Scale	3.04	0.94	-0.04	-0.73	0.96	3.00	0.86	0.01	-0.72	0.95
*ADHD*-*related Constructs*										
Adult ADHD Self-Report Scale	5.91	4.50	0.60	-0.44	0.86	7.02	4.19	0.35	-0.56	0.82
ADHD Functional Impairment	0.84	0.63	0.73	0.19	0.90	0.86	0.57	0.63	0.08	0.86
Dispositional Hyperfocus	3.44	1.10	0.01	-0.65	0.92	3.53	1.04	-0.01	-0.67	0.91

*Note. SD* = Standard Deviation

**Fig 1:**
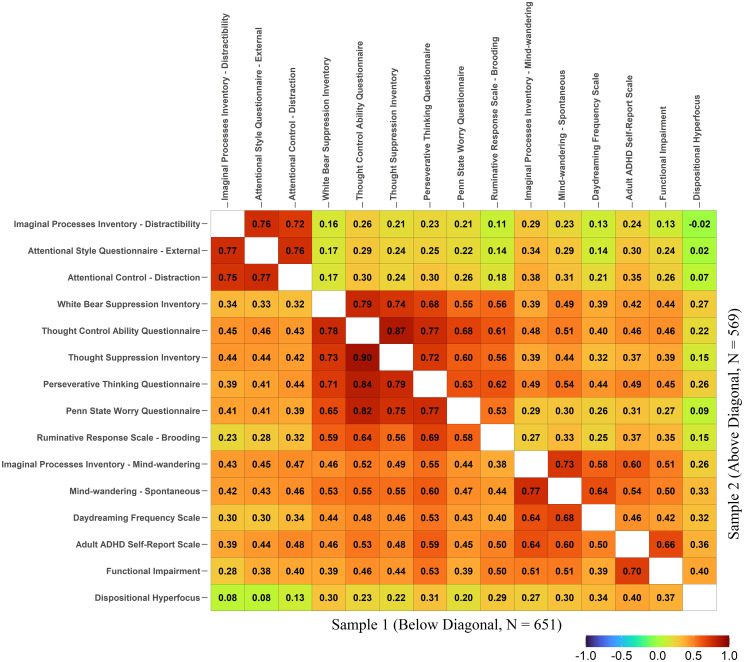
A correlation matrix for all measures. Sample 1: below the diagonal line. Sample 2: above the diagonal line.

The full correlation matrix is presented in Fig 1. The correlations for Sample 1 are presented below the diagonal, whereas those for Sample 2 are presented above the diagonal. As is clear from Fig 1, the patterns of the correlations were generally comparable for the two samples.

### Goal 1: Understanding the relationships among different facets of distractibility

#### Exploratory analyses with Sample 1

The baseline measurement model consisted of *External Distraction*, *Thought Intrusions*, *Repetitive Negative Thinking*, and *Mind-wandering*, with each factor represented by three scales. Although this model produced a good fit (*χ*^2^(48) = 157.33, *p* < .001; CFI = .98; RMSEA = .06 [.05, .08]; SRMR = .029; BIC = 14,948), we modified this model in two ways based on substantive concerns [94 p.310].

**Fig 2:**
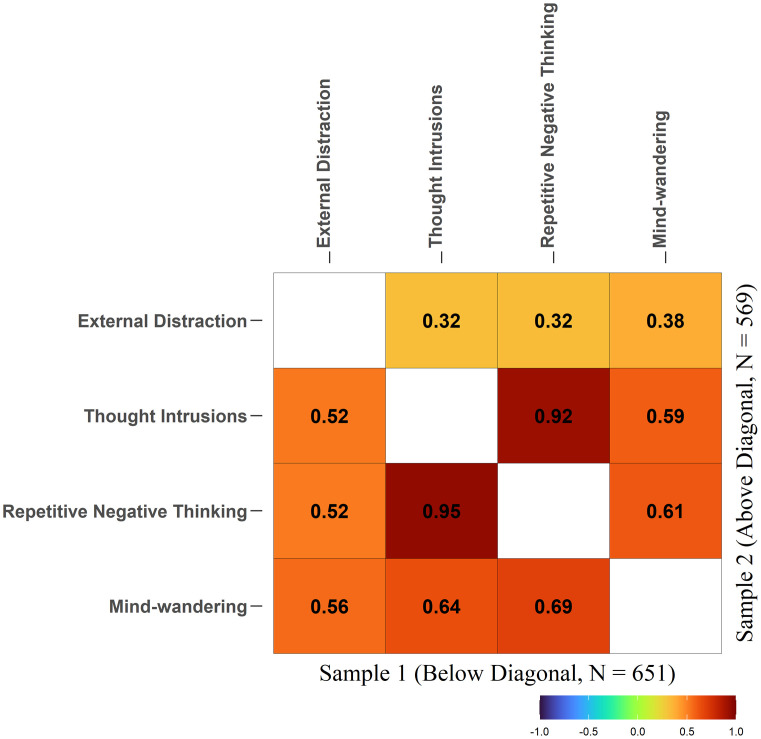
Latent correlations among external distraction, thought intrusions, repetitive negative thinking, and mind-wandering. The correlation between Thought Intrusions and Repetitive Negative Thinking was close to 1 in both samples.

First, as shown in Fig 2, the factors *Thought Intrusions* and *Repetitive Negative Thinking* had a near-perfect correlation, and they also had similar relationships with the other factors in the model (the model’s full structure can be found in S1 Fig). Thus, to avoid redundancy in the model structure, we combined *Thought Intrusions*, *Repetitive Negative Thinking* into a single factor named *Unwanted Intrusive Thoughts*. This move aligns with recommendations by Kline [94], who stressed that respecification in CFA should be guided as much as possible by substantive considerations. Given the large sample sizes of the current study and the resulting statistical power, even trivial differences may become highly significant. Here, an *r* = .95 means that the two factors shared about 85% to 90% of the variance (i.e., only ~10% and ~15% of the unique variance left). If these highly correlated constructs were kept separate, the meaning of a possible general factor would be highly biased (essentially adding a duplicate indicator). Although some details differ, some previous latent-variable analysis studies have merged some latent variables on the basis of such substantive considerations [95–98].

Second, we inspected the modification indices (MI) of the model, which suggested that allowing the residual of the TCAQ and that of the TSI to correlate with each other would produce the largest improvement in model fit (MI = 62.82). This suggestion makes sense, given that the TCAQ and the TSI shared many similarly worded items (e.g., “*There are some thoughts that enter my head without me being able to avoid it*” in the former and “*Some unwanted thoughts enter my mind without me being able to do anything about it*” in the latter; original items are available at https://osf.io/8j6p4/). Thus, we added this error covariance parameter to capture the wording similarity.

**Fig 3:**
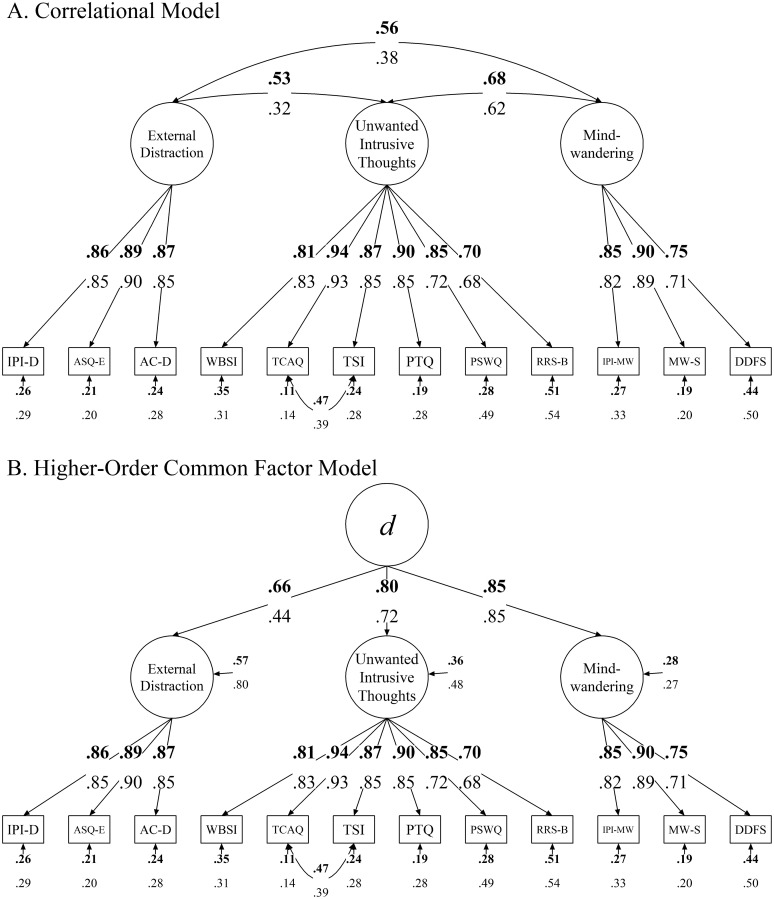
Correlational and general factor models of distractibility. Fully standardized estimates are shown, with bold fonts indicating *Sample 1* and regular fonts indicating *Sample 2*. *IPI-D*: Imaginal Processes Inventory-Distractibility; *ASQ-E*: Attentional Style Questionnaire-External; *AC-D*: Attentional Control-Distraction; *PTQ*: Perseverative Thinking Questionnaire; *PSWQ*: Penn State Worry Questionnaire; *RRS-B*: Ruminative Response Scale-Brooding; *WBSI*: White Bear Suppression Inventory; *TCAQ*: Thought Control Ability Questionnaire; *TSI*: Thought Suppression Inventory (revised); *IPI-MW*: Imaginal Processes Inventory-Mind-wandering; *MW-S*: Mind-wandering-Spontaneous; *DDFS*: Daydreaming Frequency Scale.

The resulting three-factor model is presented in Fig 3*A*. Loadings and factor correlations for this sample (Sample 1) are indicated in bold fonts. The fit of the model was good, *χ*^2^(50) = 172.73, *p* < .001; CFI = .98; RMSEA = .07 [.06, .08]; SRMR = .030; BIC = 14,953. The observed measures were generally loaded strongly onto their respective factors (*z*s >= 28.72, *p*s < .001). The three factors were also significantly correlated with each other (*r*s >= .53, *z*s >= 14.49, *p*s < .001). The model also indicated a nontrivial correlation between the error terms of the TCAQ and the TSI (*r* = .47, *z* = 9.72, *p* < .001). A re-inspection of the modification indices did not suggest further alternations that would appreciably improve model fit.

We also tested a two-factor model in which *Unwanted Intrusive Thoughts* and *Mind-wandering* were combined into one factor named *Internal Distraction*, as well as a single-factor model in which all three factors were combined into a single factor. In both models, the aforementioned error correlation between the TCAQ and the TSI was included. However, both models resulted in very poor model fit. Specifically, the fit of this two-factor model was appreciably worse compared to the three-factor model as shown in Fig 3*A*, Δ*χ*^2^(2) = 201.96, *p* < .001; BF < 1/1000 (very strong evidence against). And the fit of the single-factor model was even worse compared to the two-factor model, Δ*χ*^2^(1) = 290.00, *p* < .001; BF < 1/1000 (very strong evidence against).

The results so far indicate that *External Distraction*, *Unwanted Intrusive Thoughts*, and *Mind-wandering* were substantially correlated constructs. Therefore, we introduced a higher-order common factor *d* to capture their shared variance, as shown in Fig 3*B*. We chose the letter *d* for this general factor to echo other general factors that have been proposed in the literature, such as the general psychopathology factor *p* [99] and general intelligence factor *g* [100]. This change in model structure from correlated factors to a higher-order common factor does not alter the model fit. The first-order loadings remained the same as in the correlational model. The three first-order factors (*External Distraction*, *Unwanted Intrusive Thoughts*, and *Mind-wandering*) were all significantly loaded onto *d* (*z*s >= 19.03, *p* < .001).

To quantify the magnitude of the shared variance, we calculated the coefficient omega at Level 2 (*ω*_*L*2_), which indicates the proportion of variance among the first-order factors attributable to *d*. We found that *ω*_*L*2_ = .83, indicating that *d* explained 83% of the total variance in *External Distraction*, *Unwanted Intrusive Thoughts*, and *Mind-wandering*. The variances in the first-order factors that could not be explained by *d* were captured by their respective residual terms (.57, .36, and .28, respectively, as shown in Fig 3*B*). These residual terms were all significantly different from zero (*z*s >= 4.48, *p* < .001). Thus, although *d* captured a substantial amount of shared variance, there were still some construct-specific variances left.

We then compared the strength of the second-order loadings on *d*. To do so, we imposed a series of equality constraints on the second-order loadings of the model. Constraining all three factors to load equally on *d* substantially worsened model fit, Δ*χ*^2^(1) = 19.19, *p* < .001; BF = 1/212.43 (very strong evidence against). Thus, the three second-order loadings were not equal. To better specify the source of this non-equivalence of the *d* loadings, we conducted pairwise comparisons by constraining two of the loadings to be equal at each time and compared it to the model without the constraints. Constraining *Mind-wandering* and *External Distraction* to load equally on *d* substantially worsened model fit, Δ*χ*^2^(1) = 14.63, *p* < .001; BF < 1/1000 (very strong evidence against). Constraining *Unwanted Intrusive Thoughts* and *External Distraction* to load equally on *d* also substantially worsened model fit, Δ*χ*^2^(1) = 14.47, *p* < .001; BF = 1/26.19 (strong evidence against). But constraining *Unwanted Intrusive Thoughts* and *Mind-wandering* to load equally on *d* did not worsen model fit, Δ*χ*^2^(1) = .67, *p* = .41; BF = 16.17 (positive evidence in favor of). These results indicate that the loading of *External Distraction* on *d* was relatively weaker (albeit still significant) compared to those of *Unwanted Intrusive Thoughts* and *Mind-wandering*.

#### Replication with Sample 2

After the explorations of various models using Sample 1 as described above, we tested whether the results could be replicated using Sample 2. As shown in Fig 2, the correlation between *Thought Intrusions* and *Repetitive Negative Thinking* was again close to 1 (*r* = .92) and thus the two factors should be combined. Modification indices again suggested that adding the residual correlation between TCAQ and TSI would produce the largest improvement in model fit (MI = 31.84). Thus, adding the error covariance is also justified.

The same three-factor model as shown in Fig 3*A* resulted in a good model fit, *χ*^2^(50) = 163.24, *p* < .001; CFI = .97; RMSEA = .07 [.06, .08]; SRMR = .041; BIC = 12,526. The loadings and factor correlations for the Sample 2 data are shown in regular font below those for the Sample 1 data. Similar to Sample 1, the factor loadings (*z*s >= 24.88, *p*s < .001) and factor correlations (*r*s >= .32, *z*s >= 7.37, *p*s < .001) were all significant. Furthermore, the Sample 2 data did not support a two-factor model, Δ*χ*^2^(2) = 296.73, *p* < .001; BF < 1/1000 (very strong evidence against), or a one-factor model, Δ*χ*^2^(1) = 893.97, *p* < .001; BF < 1/1000 (very strong evidence against).

For the higher-order model as shown in Fig 3*B*, the three first-order factors were all significantly loaded onto *d* (*z*s >= 8.92, *p* < .001). The *ω*_*L*2_ of *d* was.77, indicating that *d* explained 77% of the total variance in *External Distraction*, *Unwanted Intrusive Thoughts*, and *Mind-wandering* (83% in Sample 1). In addition, the residual terms of *External Distraction*, *Unwanted Intrusive Thoughts*, and *Mind-wandering* were all significantly different from zero (*z*s >= 2.94, *p* <= .003).

Comparisons of the second-order loadings once again show that *External Distraction* was relatively weakly (albeit still significantly) loaded onto *d*. Specifically, constraining all three second-order loadings to be equal substantially worsened model fit, Δ*χ*^2^(1) = 47.95, *p* < .001; BF < 1/1000 (very strong evidence against). Constraining *Mind-wandering* and *External Distraction* to load equally on *d* substantially worsened model fit, Δ*χ*^2^(1) = 40.98, *p* < .001; BF < 1/1000 (very strong evidence against). Constraining *Unwanted Intrusive Thoughts* and *External Distraction* to load equally on *d* substantially worsened model fit, Δ*χ*^2^(1) = 28.03, *p* < .001; BF < 1/1000 (very strong evidence against). But constraining *Unwanted Intrusive Thoughts* and *Mind-wandering* to load equally on *d* did not worsen model fit, Δ*χ*^2^(1) = 1.91, *p* = .17; BF = 9.15 (positive evidence in favor of).

Overall, all of the above results based on Sample 2 were highly consistent with those based on Sample 1.

#### Measurement invariance

Next, we conducted a measurement invariance analysis to evaluate the replicability of the model at the parameter level (i.e., beyond the global model structure). Measurement invariance analysis involves testing the equivalence of models in independent samples to assure that the same constructs are being assessed in each sample. Specifically, a series of cross-group equality constraints are incrementally added to a multiple-group confirmatory factor analysis model. Four levels of invariance are typically tested: (*a*) configural invariance (i.e., equal model structure across samples), (*b*) metric invariance (i.e., equal loadings across samples), (*c*) scalar invariance (i.e., equal intercepts across samples), and (*d*) residual invariance (i.e., equal residuals across samples). If the model with new constraints does not fit appreciably worse than the model without those constraints, then the invariance hypothesis is retained.

Currently, there is no consensus on the appropriate criteria to assess invariance. We made our decisions based on multiple fit indices, including ΔCFI, ΔRMSEA, ΔSRMR, and Bayes Factors. Δ*χ*^2^ tests were not recommended as a criterion to assess invariance when the sample size is large [94]. Thus, they were reported for completeness but not considered in the assessment of invariance. All models in this analysis were identified using the effect-coding method [101].

The results, as shown in Table 3, indicate that the higher-order model passed all four levels of invariance. This indicates that the scales measured the same factors (configural) with the same unit of measurement (loadings), same origin (intercepts), and the same precision (residuals). Note that the Bayes Factor for scalar invariance was 1/2.46, indicating weak evidence against scalar invariance over metric invariance. However, considering that ΔCFI, ΔRMSEA, and ΔSRMR were all well below common cut-off values [94], we retained scalar invariance. It should be noted that metric invariance may be considered particularly important in invariance testing because it ensures that relations among the factors and measured variables are similar across samples. Overall, these results indicate that the higher-order general distractibility model was highly replicable at the parameter level.

**Table 3:** Measurement invariance analysis of the general distractibility model.

Model	Comparison	*χ* ^2^	*df*	Δ*χ* ^2^	CFI	RMSEA [90% CI]	SRMR	BF
1. configural invariance		336.18	100		.98	.07 [.06, .07]	.033	
2. metric invariance	2 vs. 1	366.11	111	28.53**	.97	.07 [.06, .07]	.045	> 1000
3. scalar invariance	3 vs. 2	446.15	122	91.76***	.97	.07 [.06, .08]	.050	1/2.46
4. residual invariance	4 vs. 3	474.52	138	28.75*	.97	.07 [.06, .07]	.050	> 1000

*Note*. The Δ*χ*^2^ column shows the Satorra-Bentler-adjusted Δ*χ*^2^ difference test, which is a function of two standard (not robust) *χ*^2^ statistics.

* *p* < .05,

** *p* < .01,

*** *p* < .001.

### Goal 2: Linking distractibility to ADHD symptoms and hyperfocus

After establishing the higher-order model of distractibility, we proceeded with our next goal: applying the model to understand individual differences in ADHD symptoms and hyperfocus. Again, we used Sample 1 for exploratory analyses and Sample 2 for replication.

#### Exploratory analyses with Sample 1

**Fig 4:**
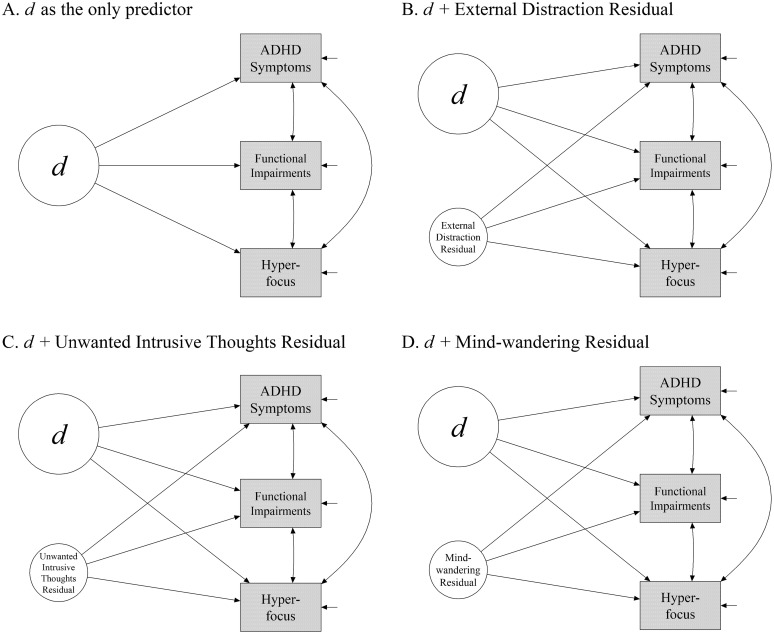
Schematic illustrations of models testing how the unity and diversity aspects of distractibility were related to ADHD symptoms, functional ADHD impairments, and hyperfocus. Panel A shows a model that tests how well *d* alone could account for variances in ADHD symptoms, functional impairments, and hyperfocus. The models in panels B-D test whether External Distraction, Unwanted Intrusive Thoughts, and Mind-wandering explain additional variance over and above d. Specifically, in Panel B, d and the non-d residual of External Distraction served as predictors. In Panel C, d and the non-d residual of Unwanted Intrusive Thoughts served as predictors. In Panel D, d and the non-d residual of Mind-wandering served as predictors. In all panels, manifest variables were not shown. The directional paths were not meant to imply causation.

First, we explored how well *d* alone could account for variances in ADHD symptoms, ADHD functional impairments, and hyperfocus. We built a model in which the three variables were simultaneously regressed onto *d*. A schematic illustration of the model is given in Fig 4*A*. The fit of the model was good, *χ*^2^(83) = 320.35, *p* < .001; CFI = .97; RMSEA = .07 [.06, .08]; SRMR = .041; BIC = 19,528. Table 4 shows the structural paths associated with *d*. As shown in the table, a higher *d* was strongly related to a higher level of ADHD symptomatology and greater functional impairments. Notably, *d* was also positively related to hyperfocus, such that a higher *d* (i.e., higher general distractibility) was associated with a *stronger* tendency to engage in hyperfocus.

**Table 4:** Standardized path coefficients of the unity and diversity components of distractibility predicting ADHD symptoms, ADHD functional impairments, and hyperfocus.

Models	*Sample* 1			*Sample* 2		
	ADHD Symptoms	Functional Impairments	Hyperfocus	ADHD Symptoms	Functional Impairments	Hyperfocus
Model A						
*d*	.78***	.66***	.36***	.74***	.67***	.37***
Model B						
*d*	.78***	.66***	.39***	.74***	.68***	.40***
External Distraction Residual	-.01	-.03	-.19***	.01	-.06	-.16***
Model C						
*d*	.80***	.65***	.35***	.74***	.65***	.38***
Unwanted Intrusive Thoughts Residual	-.07	.01	.04	-.02	.06	-.02
Model D						
*d*	.75***	.65***	.31***	.72***	.67***	.29***
Mind-wandering Residual	.11	.04	.16*	.08	.02	.22*

*Note*. The results of models A-D correspond to the models shown in Fig 4.

*p < .05,

***p < .001.

Next, we explored whether any diversity aspects of distractibility could explain any additional variance. To do so, we built models in which the three variables were regressed onto the residual variances of *External Distraction*, *Unwanted Intrusive Thoughts*, and *Mind-wandering* in addition to *d*. These residual variances were construct-specific variances in each specific form of distraction that were not explained by *d* (see Fig 3*B*). To use residual variance as a predictor, we created a latent factor to capture the residual variance in each group factor following instructions from [102]. These models are shown in Fig 4*B*–4*D*. The structural paths associated with the residual factor, therefore, test the explanatory power of construct-specific variance that is unrelated to *d*. Note that this procedure had to be conducted separately for each first-level factor because regressing on all residuals simultaneously leads to non-identification issues due to linear dependency (for a technical discussion of the issue, see Zhang et al. [103, p.536]; for a previous example of separately testing the residuals, see Berkowitz and Stern [104]).

The model shown in Fig 4*B* used the residual of *External Distraction* as an additional predictor. The model had a good fit, *χ*^2^(80) = 302.00, *p* < .001; CFI = .97; RMSEA = .07 [.06, .08]; SRMR = .036; BIC = 19,527. The key results are summarized in Table 4. *External Distraction* residual was not significantly associated with ADHD symptoms or functional impairments. A *negative* relationship emerged between the residual of *External Distraction* and hyperfocus, such that a higher susceptibility to external distraction was associated with a weaker hyperfocus tendency after controlling for *d*.

The model shown in Fig 4*C* used the residual of *Unwanted Intrusive Thoughts* as an additional predictor. The model had a good fit, *χ*^2^(80) = 316.49, *p* < .001; CFI = .97; RMSEA = .07 [.06, .08]; SRMR = .040; BIC = 19,542. The results, summarized in Table 4, show that *Unwanted Intrusive Thoughts* residual did not significantly explain additional variance in any of the variables.

The model shown in Fig 4*D* used the residual of *Mind-wandering* as an additional predictor. The fit of the model was good, *χ*^2^(80) = 316.06, *p* < .001; CFI = .97; RMSEA = .07 [.06, .08]; SRMR = .040; BIC = 19,539. The results, summarized in Table 4, show that *Mind-wandering* residual was not significantly associated with ADHD symptoms or functional impairments. There was a *positive* relationship with hyperfocus, such that a higher susceptibility to mind-wandering was associated with a stronger tendency to engage in hyperfocus even after controlling for *d*.

Overall, our analyses using Sample 1 show that *d* was the dominant predictor of ADHD symptoms and functional impairments. When the residual factors were added, there were some minor fluctuations in the effects of *d*, but *d* still remained a highly significant predictor. In contrast, the residuals of *External Distraction*, *Unwanted Intrusive Thoughts*, and *Mind-wandering* did not explain meaningful variance in ADHD symptoms and functional impairments.

The results for hyperfocus were more complex. While *d* was also positively associated with hyperfocus, the residuals of *External Distraction* and *Mind-wandering* also explained additional variance. Interestingly, when controlling for *d*, increased susceptibility to external distraction was associated with a weaker tendency to engage in hyperfocus, whereas increased susceptibility to mind-wandering was associated with a stronger tendency to engage in hyperfocus. This contrasting pattern of results will be discussed in more depth later in the Discussion section.

#### Replication with Sample 2

All the results for Sample 1 were replicated in the Sample 2 data (see Table 4). First, the model with *d* as the only predictor (Fig 4*A*) had a good fit, *χ*^2^(83) = 258.75, *p* < .001; CFI = .97; RMSEA = .06 [.06, .07]; SRMR = .046; BIC = 16,639. A higher *d* was associated with a higher level of ADHD symptomatology, greater functional impairments, and a stronger hyperfocus tendency.

Second, the model with the residual of *External Distraction* as an additional predictor (Fig 4*B*) had a good fit, *χ*^2^(80) = 242.69, *p* < .001; CFI = .97; RMSEA = .06 [.05, .07]; SRMR = .042; BIC = 16,641. *External Distraction* residual was not significantly associated with ADHD symptoms and functional impairments, but it was negatively and significantly associated with hyperfocus.

Third, the model with the residual of *Unwanted Intrusive Thoughts* as an additional predictor (Fig 4*C*) had a good fit, *χ*^2^(80) = 255.23, *p* < .001; CFI = .97; RMSEA = .07 [.06, .07]; SRMR = .046; BIC = 16,654. *Unwanted Intrusive Thoughts* residual did not significantly explain additional variance in any of the variables.

Finally, the model with the residual of *Mind-wandering* as an additional predictor (Fig 4*D*) also had a good fit, *χ*^2^(80) = 252.46, *p* < .001; CFI = .97; RMSEA = .07 [.06, .07]; SRMR = .044; BIC = 16,651. *Mind-wandering* residual was not significantly associated with ADHD symptoms and functional impairments, but it was positively and significantly associated with hyperfocus.

#### Additional analyses

We also conducted two additional analyses, both of which can be found in S2 File. First, the relationship between *d* and hyperfocus was relatively weaker compared to ADHD symptoms and functional impairments (Supplemental Analysis 1 in S2 File). Second, while *d* was substantially associated with both inattention symptoms (e.g., having difficulty keeping attention) and hyperactive/impulsive symptoms (e.g., fidgeting or squirming with hands or feet), it was more strongly associated with inattention symptoms (Supplemental Analysis 2 in S2 File). None of the residual factors explained meaningful variance. Both analyses were replicated in Sample 2.

## Discussion

The current study aimed to understand the factor structure of distractibility. Using a latent modeling approach, we assessed how individual differences in self-reported vulnerability to several forms of real-world distraction were related. Based on the pattern of the correlations, we developed a latent-variable model that incorporates a higher-order common factor to capture individual differences in general distractibility. We then examined how this higher-order model can account for individual differences in ADHD behavioral symptoms, ADHD functional impairments, and hyperfocus.

With two large samples, we conducted exploratory analyses using the first sample and an internal replication using the second sample. It is worth noting that the characteristics of the two samples were substantially different in terms of age (26.9 ± 5.0 in Sample 1 vs. 18.8 ± 1.2 in Sample 2), gender distribution (45.3% female in Sample 1 vs. 66.3% female in Sample 2), racial composition (67.7% white in Sample 1 vs. 56.8% white in Sample 2), and occupation (33.9% college students vs. 100% college students). Despite these differences, all key results in Sample 1 were successfully replicated in Sample 2, thus providing solid empirical ground for the following discussion of results.

### Goal 1: Understanding the relationships among different facets of distractibility

Whereas the term “distraction” is commonly used to describe how our attention is diverted by both internal (e.g., our own thoughts) and external (e.g., pop-up advertisements) sources in daily life, the study of distraction has focused heavily on the latter. Although other fields have studied phenomena such as mind-wandering and intrusive thoughts, there has been relatively little integration between them until recently [55].

Extending previous findings, a critical finding of our study is the identification of a higher-order factor that could be construed to represent a general distractibility trait. Indeed, we found that there was very much in common among external distraction, unwanted intrusive thoughts, and mind-wandering: *d* accounted for about 80% of the total variance in the three factors (83% in Sample 1 and 77% in Sample 2). A measurement invariance analysis further showed generalizability of the higher-order model at the parameter level. Taken together, our findings underscore the notable shared variance among several distractibility-related constructs, suggesting a reliable trait reflecting individual differences in (perceived) distractibility.

### Goal 2: Linking distractibility to ADHD symptoms and hyperfocus

#### ADHD symptoms and associated functional impairments

In Goal 2 of the current study, we related our higher-order model of distractibility to individual differences in self-reported ADHD symptoms and functional impairments. Across two samples, we found that *d* was substantially related to individual differences in ADHD symptoms and functional impairments. Furthermore, *d* was more related to the inattentive symptoms than to the hyperactive/impulsive symptoms (see Supplemental Analysis 2 in S2 File). These results further speak to the validity of *d* as a measure of general distractibility.

These results also have important implications for the nature of the heightened distractibility in ADHD. Existing research on distractibility and ADHD has often examined specific facets of distractibility in isolation and thereby neglected the substantial overlap among different facets of distractibility. When ADHD is simultaneously associated with vulnerability to multiple forms of distraction, it may be more parsimonious to view these associations as reflecting a single association with general distractibility rather than separate associations with each specific form of distractibility. In fact, our results show that once *d* was included in the model, none of the residual factors explained variance in ADHD symptoms above and beyond *d*. In particular, *External Distraction* did not explain any meaningful variance in ADHD symptoms and functional impairments beyond *d* despite its substantial construct-specific variance. Thus, the results indicate that the heightened distractibility in ADHD symptomatology is best characterized by general distractibility instead of by any single type of distraction alone.

#### Hyperfocus

Our findings provide insight into the nature of hyperfocus. We discovered that individuals who were more generally distractible tended to report *more* frequent hyperfocus episodes. This counterintuitive relationship implies that focused and distracted attention might share some underlying features. One possible interpretation is that individuals often hyperfocus on the same subjects that distract them from more critical tasks. For example, someone might hyperfocus on watching TV, which serves as a distraction from their work assignments. Alternatively, distractibility and hyperfocus might both be indicative of deficits in attentional control. Consequently, individuals who are easily distracted by irrelevant information may also tend to hyperfocus on certain subjects because they have difficulties controlling when they should focus attention and when they should disengage attention.

Interestingly, hyperfocus was explained by other predictors besides *d*. When *d* was controlled for, hyperfocus was positively related to mind-wandering but was negatively related to external distraction (see Table 4). What might explain this contrasting pair of relationships?

Current theories of mind-wandering consider perceptual decoupling, or reduced processing of immediate perceptual input, as a unique feature of mind-wandering [35, 105]. Both behavioral and neurological evidence indicates that mind-wandering is associated with reduced processing of external information and events [35]. It has been suggested that perceptual decoupling is critical for mind-wandering to unfold because it protects the internal train of thought from external disruptions [12]. Similarly, many descriptions of hyperfocus experiences include common qualities such as distorted time perception, failure to attend to the world, ignoring personal needs, feelings of total engrossment, etc. [13]. Therefore, it seems plausible that a decoupling process may be involved in hyperfocus to reduce the processing of information that is not related to the current hyperfocus subject. This potential similarity between hyperfocus and mind-wandering may also explain why external distraction was negatively related to hyperfocus once *d* was accounted for: those who are more sensitive to sensory information may have more difficulties immersing themselves in a hyperfocus state.

In sum, whereas ADHD symptoms mostly reflect heightened general distractibility, hyperfocus appears to be a more complex construct that displays qualities of both distraction (in terms of its association with *d*) and concentration (in terms of decoupling oneself from subjects unrelated to the hyperfocus content). These results suggest the utility of considering diversity aspects of distractibility in explaining other variables of interest.

### Limitations and future directions

As the first large-scale latent-variable study of its kind, the current study goes substantially beyond previous studies reviewed earlier [54, 55, 57] that tried to make a case for a general distractibility factor. Moreover, the current study also related the *d* factor to two ADHD-related variables (ADHD symptoms and hyperfocus). Despite these substantial contributions to the literature, the study is also limited in a number of ways. We will discuss two in detail here.

#### Alternative interpretations of what *d* represents

One limitation of the current study is that, although the results reported above demonstrate that it is possible to statistically extract a general *d* factor, a latent-variable analysis like the one we conducted, by itself, does not specify what the *d* factor really represents as a psychological construct. As such, the current study leaves open the possibility that the *d* factor can be accounted for—at least in part, if not entirely—by other psychological constructs. Note that this interpretational ambiguity of a general factor is not unique to the current study; in fact, it is also applicable to other latent-variable studies postulating a general factor, such as the *g* factor for intelligence [100], the *p* factor for psychopathology [99], and the Common EF factor for executive functions [47, 48].

Nevertheless, two alternative interpretations–one methodological and one psychological–need to be considered in the context of the current study.

One such possibility is that the *d* factor might be a methodological artefact in that most of its variance can be explained by substantial method variance attributable to response biases of some sort, such as social desirability [106] or a tendency to complain about cognition [107]. Given that the current study focused on a large number of self-report measures, we cannot completely rule out such a possibility. Nevertheless, we suspect that response biases, by themselves, are unlikely to be able to account for substantial shared variance among the measures administered in the current study for a number of reasons. First, the questionnaires used in the current study are well-validated, with many of them being used extensively in both research and clinical settings. Second, the current study was conducted online, with participants completing the questionnaires anonymously, without facing any experimenter in person, which presumably reduces the likelihood that participants’ responses are strongly affected by at least one such potential bias-related factor, social desirability. Finally, it is difficult to test a response-bias account of the *d* factor, given that it is not necessarily clear what specific response biases might be operating that are strong enough to affect participants’ responses to all of the questionnaire measures. Thus, it is unlikely that the *d* factor is a pure methodological artefact reflecting unspecified method variance of some sort.

A second alternative interpretation that needs to be considered is that the *d* factor can be substantially explained by some other psychological constructs. One interesting possibility is that the *d* factor can be substantially (if not entirely) accounted for by a Big Five personality trait, neuroticism [108]. Given that neuroticism is associated with one’s tendency to experience negative affects, such as anger, anxiety, and depression [109], it is likely that individual differences in neuroticism substantially overlaps with the distractibility constructs examined in the current study that are clearly linked to negative affect (i.e., the unwanted intrusive thoughts factor that encompasses the repetitive negative thinking and intrusive thoughts factors). What is not clear is to what extent neuroticism is related to the other two distractibility constructs less directly associated with negative affect, external distraction, and mind-wandering. As shown in the higher-order common factor model (Fig 3*B*), however, the *d*-factor loadings are not necessarily dominated by the unwanted intrusive thoughts factor. Moreover, it is generally the case that the magnitudes of correlations with neuroticism are substantially higher for repetitive negative thinking and intrusive thoughts [110–112] than for mind-wandering and external distraction [113–117]. Thus, neuroticism, conceptualized as a psychological trait, may not be able to fully explain the *d* factor.

Because we did not include a measure of neuroticism in the current study, it is not possible to precisely specify how much of the variance in the *d* factor can be accounted for by neuroticism. However, just as Friedman et al. [118] were able to demonstrate that general intelligence or IQ accounted for only half (~50%) of the variation in a general executive function factor (or Common EF), future studies should be able to quantify the extent to which neuroticism and other general factors can explain the variance in *d*.

More broadly, the effort to characterize what the *d* factor represents psychologically will likely be facilitated by systematic attempts to examine the relationship between *d* and other psychological constructs that tap into attention and cognitive control, such as working memory capacity, general fluid intelligence, executive function, and attentional control, given that all these constructs have been theorized to play an overarching role in maintaining task focus and resisting distraction interference [48, 49, 51, 119, 120]. Also, given the prominent role of distractibility in various psychiatric disorders, it might be worthwhile examining the relationship between *d* and the general psychopathology factor *p* [99].

#### Generalizability of the current results to behavioral measures of distractibility

As a first step toward achieving a comprehensive understanding of the relationships among different facets of distractibility, the present study conducted a latent-variable analysis focusing on self-report questionnaires only. Given a strong need for a comprehensive assessment of several distractibility-related constructs simultaneously in a methodologically rigorous way (e.g., latent-variable analysis), we believe that our focus on self-report measures in this study is fully justified, especially in the light of the state of the literature on the notion of a general distractibility factor [54, 55, 57].

Having said that, however, we are also fully aware that there are other ways of assessing different facets of distractibility, such as behavioral tasks, experience-sampling in the laboratory as well as in more naturalistic everyday settings, and even cognitive neuroscience measures (e.g., EEG/ERP, neuroimaging). Thus, another important limitation of the current study is that we did not include any such non-questionnaire-based ways of assessing different facets of distractibility that would have helped us evaluate whether our findings (especially regarding the *d* factor) can be generalized to other assessment methods. Given that two of the distractibility-related constructs examined here–external distraction and mind-wandering–have been studied with multiple methods, relating the current results to those based on those other assessment methods (especially behavioral tasks) seems to be an important and necessary next step. In this regard, we mention two important considerations here.

First, the current state of the field is such that the availability of well-established objective behavioral measures (e.g., based on RTs or error rates) is limited, especially for more clinically oriented constructs like intrusive thoughts and repetitive negative thinking. Even when there are some behavioral tasks available, their psychometric properties have been found suboptimal [121]. For example, Meier [56] demonstrated that behavioral measures of external distraction developed earlier [54, 55] had an internal consistency of.08—.26, which seems insufficient to reliably capture individual differences in external distractibility. Moreover, some behavioral measures of one’s ability to filter out external distractions (e.g., flanker and antisaccade) do not correlate well with each other, leading some researchers to suggest that individual differences in interference-control abilities as assessed by these measures are task-specific [122]. Thus, it currently seems difficult to conduct a large-scale latent-variable study of the sort reported here that examines multiple distractibility-related constructs simultaneously using multiple measures for each construct not only for questionnaires but also for behavioral tasks. Further task development and validation seem necessary before such a study can be conducted in a rigorous, psychometrically sound manner.

Second, it has become increasingly clear that different ways of assessing the same distractibility-related construct (e.g., mind-wandering) may not necessarily correlate substantially with each other [123, 124]. For example, Kane et al. [124, p.1278] recently demonstrated that mind-wandering, as measured by thought probes in laboratory settings, is not correlated with mind-wandering in real life, as measured with experience-sampling, leading them to conclude that “any relation between laboratory and overall daily-life mind-wandering propensities is not robust”.

More generally, the relationship (or lack thereof) between questionnaires and behavioral measures has become an important issue that goes beyond the measurement of distractibility [117, 125], especially in two research domains closely related to distractibility: executive functions [126] and self-control [75]. Thus, it is quite possible that the current results based on questionnaires may not generalize to those based on behavioral measures.

We should hasten to emphasize here, however, that the potentially limited generalizability of the questionnaire-based results to other assessment methods does not negate the significance of the current results. In fact, an emerging view is that self-report ratings and behavioral tasks may both capture important but different aspects of the same construct, making them complementary to each other [75, 76, 127]. For example, according to a recent framework [127], the *d* factor, as measured by questionnaires, may capture individual differences in the control of distraction that (a) is involved in everyday life situations (e.g., attending lectures), (b) is often associated with emotional content (e.g., controlling worrying thoughts), (c) reflects metacognitive awareness (raters needed to have some insight into their abilities), and (d) involves strategic control over the environments (e.g., one might intentionally muting cellphones while working to avoid distractions). These aspects of distraction control are not captured by laboratory-based behavioral measures that are associated with clear, well-defined task goals, but are emotionally neutral and devoid of rich everyday contexts.

Overall, an important future challenge toward achieving a comprehensive understanding of the nature and structure of individual differences in distractibility is not only to consider multiple facets of distractibility simultaneously but also to develop a unified framework that can synthesize potentially different patterns of results we may see for different ways of assessing distractibility.

## Supporting information

S1 Fig The structure of the baseline measurement model. (PNG)

S1 File Summary of fit indices. (PDF)

S2 File Additional analyses on ADHD symptoms and hyperfocus. (PDF)
