# Correction: A *d* factor? Understanding trait distractibility and its relationships with ADHD symptomatology and hyperfocus

**DOI:** 10.1371/journal.pone.0332258

**Published:** 2025-09-11

**Authors:** Han Zhang, Akira Miyake, Jahla Osborne, Priti Shah, John Jonides

The Funding statement for this article is incorrect. The correct Funding statement is as follows: This work was supported by the National Science Foundation [grant number: 2238151] awarded to the University of Michigan with JJ as Principal Investigator and the National Institute of Mental Health (Unique Federal Award Identification Number (FAIN): R21MH129909) awarded to the University of Michigan with JJ as Principal Investigator. The funders had no role in study design, data collection and analysis, decision to publish, or preparation of the manuscript.
