# Supplementary figures and images for: A d factor? Understanding trait distractibility and its relationships with ADHD symptomatology and hyperfocus
Source: PLoS One. 2023 Oct 25;18(10):e0292215. doi: 10.1371/journal.pone.0292215 (PMC10599552; doi:10.1371/journal.pone.0292215)

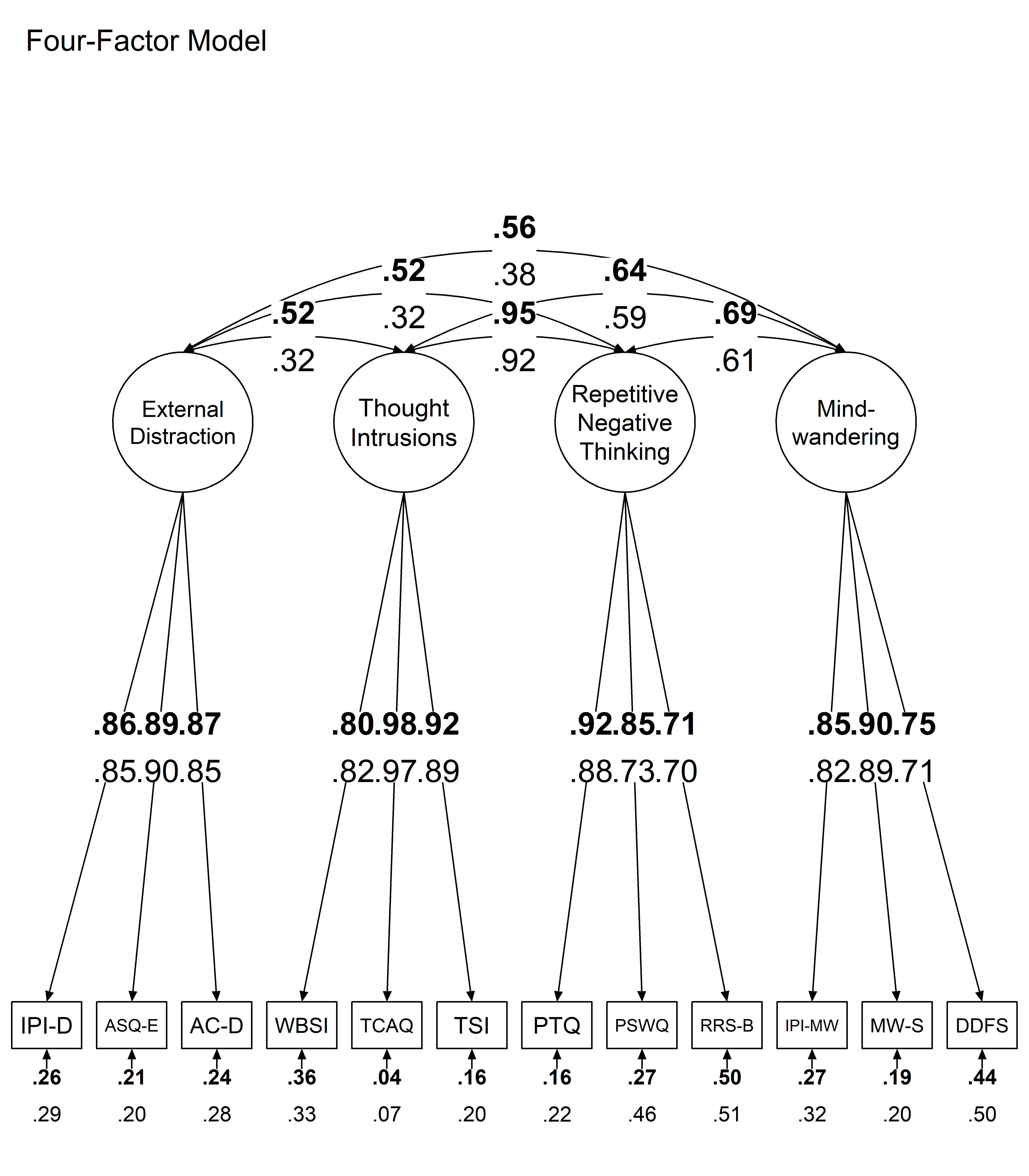

Supplement: S1 Fig — (PNG) [file pone.0292215.s001.png]
