# Supplementary material for: A d factor? Understanding trait distractibility and its relationships with ADHD symptomatology and hyperfocus
Source: PLoS One. 2023 Oct 25;18(10):e0292215. doi: 10.1371/journal.pone.0292215 (PMC10599552; doi:10.1371/journal.pone.0292215)
Supplement: S1 File — (PDF) [file pone.0292215.s002.pdf]

Fit Indices for Models in the Main Text

Contents

Table 1: Fit Indices for Models in the Main Text

Goal	Model	S-B χ^2	*df*	CFI	RMSEA [90% CI]	SRMR	BIC
Sample 1							
Goal 1	4 Factors	157.33	48	.98	.06 [.05, .08]	.029	14,948
Goal 1	3 Factors	172.73	50	.98	.07 [.06, .08]	.030	14,953
Goal 1	2 Factors	636.03	52	.89	.15 [.14, .16]	.079	15,526
Goal 1	1 Factor	1,351.51	53	.75	.22 [.21, .23]	.122	16,457
Goal 1	Higher Order	172.73	50	.98	.07 [.06, .08]	.030	14,953
Goal 2	*d* as the only predictor	320.35	83	.97	.07 [.06, .08]	.041	19,528
Goal 2	*d* and External Distraction residual as predictors	302.00	80	.97	.07 [.06, .08]	.036	19,527
Goal 2	*d* and Unwanted Intrusive Thoughts residual as predictors	316.49	80	.97	.07 [.06, .08]	.040	19,542
Goal 2	*d* and Mind-wandering residual as predictors	316.06	80	.97	.07 [.06, .08]	.040	19,539
Sample 2							
Goal 1	4 Factors	143.58	48	.98	.06 [.05, .07]	.040	12,516
Goal 1	3 Factors	163.24	50	.97	.07 [.06, .08]	.041	12,526
Goal 1	2 Factors	583.14	52	.87	.14 [.13, .15]	.090	12,997
Goal 1	1 Factor	1,401.78	53	.68	.23 [.22, .24]	.152	13,924
Goal 1	Higher Order	163.24	50	.97	.07 [.06, .08]	.041	12,526
Goal 2	*d* as the only predictor	258.75	83	.97	.06 [.06, .07]	.046	16,639
Goal 2	*d* and External Distraction residual as predictors	242.69	80	.97	.06 [.05, .07]	.042	16,641
Goal 2	*d* and Unwanted Intrusive Thoughts residual as predictors	255.23	80	.97	.07 [.06, .07]	.046	16,654
Goal 2	*d* and Mind-wandering residual as predictors	252.46	80	.97	.06 [.06, .07]	.044	16,651
