# Supplementary material for: A d factor? Understanding trait distractibility and its relationships with ADHD symptomatology and hyperfocus
Source: PLoS One. 2023 Oct 25;18(10):e0292215. doi: 10.1371/journal.pone.0292215 (PMC10599552; doi:10.1371/journal.pone.0292215)
Supplement: S2 File — (PDF) [file pone.0292215.s003.pdf]

# Model Fit Information and Additional Analyses

## Contents

**1 Additional Analysis 1: Comparing the Explanatory Power of *d* on ADHD Symptoms, Functional Impairments, and Hyperfocus** **1**
1.1 Sample 1 . . . . . . 1
1.2 Sample 2 . . . . . . 1

**2 Additional Analysis 2: Separately Predicting Inattentive Symptoms and Hyperactive/Impulsive Symptoms** **2**
2.1 Sample 1 . . . . . . 2
2.2 Sample 2 . . . . . . 3

# 1 Additional Analysis 1: Comparing the Explanatory Power of *d* on ADHD Symptoms, Functional Impairments, and Hyperfocus

## 1.1 Sample 1

To compare how strongly $d$ was related to these variables, we imposed equality constraints on the structural paths of $d$ in the model demonstrated in Figure 4A of the main text. When all three paths were constrained to be equal, model fit was appreciably worse, $\Delta\chi^2(2) = 85.82$, $p < .001$; BF $< 1/1000$ (very strong evidence against). This indicates that $d$ was not equally associated with these variables. Then, we conducted pairwise comparisons by constraining two of the paths to be equal at each time and compared it to the model without the constraints. Constraining $d$ to equally relate to ADHD symptoms and hyperfocus led to appreciably worse fit, $\Delta\chi^2(1) = 88.60$, $p < .001$; BF $< 1/1000$ (very strong evidence against). Constraining $d$ to equally relate to functional impairments and hyperfocus also led to appreciably worse fit, $\Delta\chi^2(1) = 30.62$, $p < .001$; BF $< 1/1000$ (very strong evidence against). Constraining $d$ to equally relate to ADHD symptoms and functional impairments again led to appreciably worse fit, $\Delta\chi^2(1) = 15.87$, $p < .001$; BF $= 1/27.40$ (strong evidence against). These results indicate that, among the three variables, $d$ had the strongest relationship with ADHD symptoms and the weakest (albeit still significant) association with hyperfocus.

## 1.2 Sample 2

We compared the strength of these associations in the same way as in the Sample 1 analysis. Constraining all three paths to be equal led to appreciably worse fit, $\Delta\chi^2(2) = 54.55$, $p < .001$; BF $< 1/1000$ (very strong evidence against). Thus, the three paths were not equal in strength. Constraining $d$ to equally relate to ADHD symptoms and hyperfocus led to appreciably worse fit, $\Delta\chi^2(1) = 62.34$, $p < .001$; BF $< 1/1000$ (very strong evidence against). Constraining $d$ to equally relate to functional impairments and hyperfocus also led to appreciably worse fit, $\Delta\chi^2(1) = 32.98$, $p < .001$; BF $< 1/1000$ (very strong evidence against). Different from the results of Sample 1, constraining $d$ to equally relate to ADHD symptoms and functional impairments did not appreciably worsen model fit, $\Delta\chi^2(1) = 3.61$, $p < .001$; BF $= 4.83$ (positive evidence in favor of). These results were generally consistent with those of Sample 1 by showing that $d$ had a weaker relationship with hyperfocus compared to ADHD symptoms and functional impairments.

# 2 Additional Analysis 2: Separately Predicting Inattentive Symptoms and Hyperactive/Impulsive Symptoms

## 2.1 Sample 1

ADHD symptomatology is typically considered to be comprised of two distinct but related groups of symptoms: inattention symptoms (e.g., having difficulty keeping attention) and the hyperactive/impulsive symptoms (e.g., fidgeting or squirming with hands or feet). In the Adult ADHD Self-report Scale, each group of symptoms was measured by 9 items. The inattentive subscale had a mean of 3.46 (SD = 2.65) and an $\alpha$ of .80. The hyperactive/impulsive subscale had a mean of 2.45 (SD = 2.32) and an $\alpha$ of .77. The two subscales had a correlation of .64, $p < .001$.

We used $d$ and non-$d$ residuals to predict the two symptom groups separately. These models are illustrated in Figure S1.

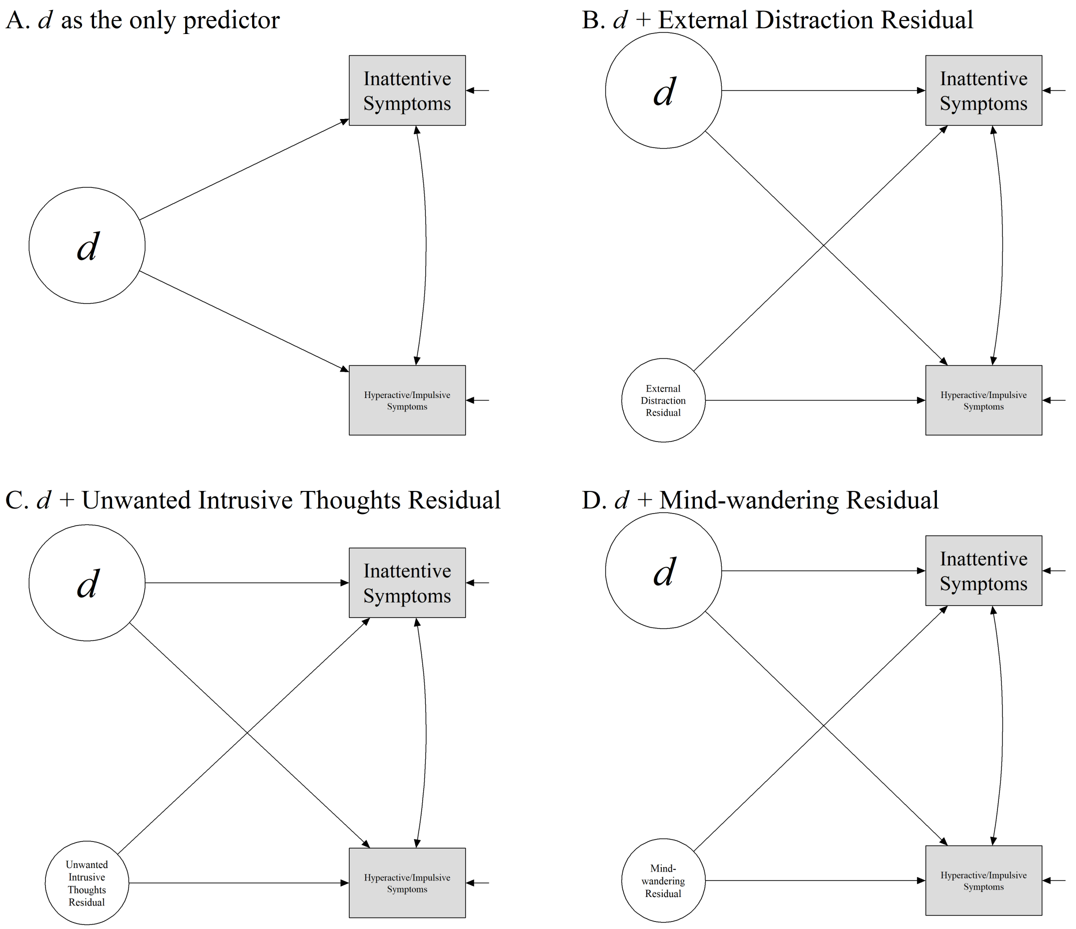


Figure S1: Schematic illustrations of models testing how the unity and diversity aspects of distractibility were related to inattentive symptoms and hyperactive/impulsive symptoms.

All models had an acceptable global fit. The key results, as shown in Table S1, indicate that $d$ was the predominant predictor of both symptom groups; none of the residual factors explained additional variance.

We also compared how well $d$ explained inattentive and hyperactive/impulsive symptoms in the model shown in panel A of the figure above. We did so by imposing equality constraints on the two paths of $d$ and compared the fit of the constrained model with the fit of the one without the constraints. Imposing $d$ to

Table S1: Standardized path coefficients of the unity and diversity components of distractibility predicting inattentive and hyperactive/impulsive symptoms of ADHD.

| | *Sample* 1 | | *Sample* 2 | |
|---|---|---|---|---|
| Models | Inattentive | Hyperactive/Impulsive | Inattentive | Hyperactive/Impulsive |
| Model A | | | | |
| *d* | .77*** | .62*** | .69*** | .60*** |
| Model B | | | | |
| *d* | .77*** | .63*** | .68*** | .60*** |
| External Distraction Residual | .02 | -.05 | .07 | -.04 |
| Model C | | | | |
| *d* | .80*** | .64*** | .74*** | .60*** |
| Unwanted Intrusive Thoughts Residual | -.09 | -.05 | -.14 | .02 |
| Model D | | | | |
| *d* | .75*** | .59*** | .65*** | .61*** |
| Mind-wandering Residual | .09 | .11 | .11 | .00 |

*Note.* The results of models A-D correspond to the models shown in Figure S1. *$p$ < .05, ***$p$ < .001.

equally predict inattentive and hyperactive/impulsive symptoms substantially worsened model fit, $\Delta\chi^2(1) = 98.49$, $p < .001$; BF < 1/1000 (very strong evidence against). Therefore, $d$ was more strongly associated with inattentive symptoms than with hyperactive/impulsive symptoms.

## 2.2 Sample 2

The inattentive subscale had a mean of 3.94 (SD = 2.50) and an $\alpha$ of .74. The hyperactive/impulsive subscale had a mean of 3.08 (SD = 2.31) and an $\alpha$ of .73. The two subscales had a correlation of .52, $p < .001$.

We fit the same models as shown in Figure S1. All models had an acceptable global fit. The key results, as shown in Table S1, replicated those of Sample 1: $d$ was the predominant predictor of both symptom groups; none of the residual factors explained additional variance.

We again compared how well $d$ explained inattentive and hyperactive/impulsive symptoms. Imposing $d$ to equally predict inattentive and hyperactive/impulsive symptoms slightly worsened model fit, $\Delta\chi^2(1) = 9.89$, $p = .002$; BF = 1/3.61 (positive evidence against). These results were generally consistent with those of Sample 1 by showing that $d$ was more strongly associated with inattentive symptoms than with hyperactive/impulsive symptoms.
